# A Modification of the Newborn Operation for Cloacal Exstrophy: Leaving the Cecal Plate Untouched

**DOI:** 10.1055/s-0044-1791814

**Published:** 2024-10-21

**Authors:** Elizaveta Bokova, Shimon E. Jacobs, Laura Tiusaba, Christina P. Ho, Briony K. Varda, Hans G. Pohl, Christina Feng, Victoria A. Lane, Caitlin A. Smith, Andrea T. Badillo, Richard J. Wood, Marc A. Levitt

**Affiliations:** 1 Division of Colorectal and Pelvic Reconstruction, Children's National Hospital, Washington, District of Columbia, United States; 2 Department of Surgery, Children's National Hospital, Washington, District of Columbia, United States; 3 Department of Surgery, Colorectal and Pelvic Reconstructive Surgery, Children's National Hospital, Washington, District of Columbia, United States; 4 Department of Urology, Children's National Hospital, Washington, District of Columbia, United States; 5 Department of Urology, Children's National Hospital, Washington, District of Columbia, United States; 6 Department of Paediatric Surgery, Great North Children's Hospital, Newcastle Upon Tyne, United Kingdom; 7 Department of Pediatric and Thoracic General Surgery, Seattle Children's Hospital, Seattle, Washington, United States; 8 Department of Colorectal and Pelvic Reconstruction, Children's National Hospital, Washington, District of Columbia, United States; 9 Department of Pediatric Colorectal and Pelvic Reconstructive Surgery, Nationwide Children's Hospital, Columbus, Ohio, United States

**Keywords:** cloaca, cloacal exstrophy, cecum, bladder augmentation, reconstruction

## Abstract

The conventional approach to managing a newborn with cloacal exstrophy typically includes separating the cecal plate from between the two hemibladders, tubularizing it to be included in the fecal stream, creating an end colostomy, and bringing the two bladder halves together. This study introduces an alternative approach wherein the cecal plate is retained in its original position and designated for future use as an autoaugment of the bladder. Four cases of cloacal exstrophy cases managed between November 2019 and February 2024 are described, with surgical approach and postoperative outcomes reported. Two patients who underwent traditional reconstruction experienced bacterial overgrowth attributed to stasis in the cecal plate, which manifested in increased ostomy output and feeding intolerance. Treatment in these two cases was to remove the cecum from the fecal stream and use it instead for a bladder augment. Learning from these cases, the third and fourth newborn's approach involved retaining the cecum in situ for autoaugmentation of the bladder and performing an ileal to hindgut anastomosis. No postoperative acidosis occurred in these patients. The alternative approach to the newborn management of cloacal exstrophy whereby the cecal plate is left in situ can decrease stasis and postoperative bacterial overgrowth. It allows for an autoaugmentation of the bladder and is technically easier than the traditional rescue of the cecal plate from within the two hemibladders.

## Introduction

Cloacal exstrophy, a rare condition diagnosed in approximately 1 in 320,000 live births, 1 has undergone a significant evolution in its surgical treatment development. 2 The traditional management described in the literature involves excising the cecal plate from between the two hemibladders, tubularizing it, incorporating it into the fecal stream, creating an end colostomy, and reapproximating together the two bladder halves now with the cecum removed from within them. 3

The goal for the newborn operation is to maximize colonic length, with the concept being that saving the cecum for the fecal stream would be beneficial and would remove the risk of urinary absorption by the cecum leading to acidosis. 4 5 6 However, several cases the authors experienced led to a reconsideration of this strategy. The patulous cecum in the fecal stream led to bacterial overgrowth and prompted its removal from the fecal stream and use of it instead for bladder augmentation. This maneuver eliminated the bacterial overgrowth and prompted the idea that the cecal plate should be left within the two hemibladders at the newborn operation.

Based on our experience with the traditional surgical approach that resulted in suboptimal outcomes in the long term in two cases, we propose a surgical strategy for the newborn cloacal exstrophy repair that avoids removing the cecal plate and tubularizing it and instead leaving it behind as an autoaugment of the bladder. We hypothesized that this approach would reduce the risk of stasis- and inflammation-related complications such as bacterial overgrowth and excessive stomal output compared to the traditional technique, would be technically easier and advantageous for later bladder closure, and would not lead to problematic acidosis related to urinary absorption by the colonic mucosa.

## Case Reports

### Case 1

**Fig. 1:**
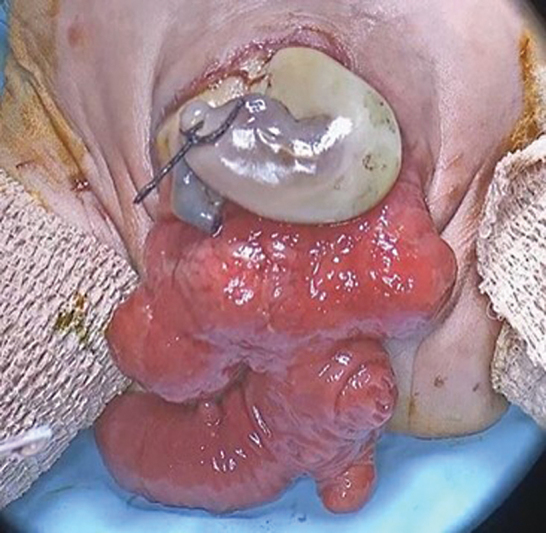
Cloacal exstrophy. (This image is provided courtesy of the Children's National Hospital archives.)

A 4-year-old girl with cloacal exstrophy ( Fig. 1 ) was initially managed with an ileostomy creation in the newborn period. She underwent reconstruction according to the traditional treatment protocol, which included hindgut rescue, cecal plate tubularization, and end colostomy creation. After the procedure, the patient was repeatedly admitted to the intensive care unit for recurrent episodes of bacteremia and obstructive symptoms. Additionally, she experienced intolerance to feeds resulting in dependence on parenteral nutrition.

An exploratory laparotomy at the age of 5 years revealed a dilated and redundant cecum. This spherical structure was removed from the fecal stream with an anastomosis created between the terminal ileum and distal hindgut. The isolated cecum was then brought to the abdominal wall, irrigated via the appendix, and ultimately used for bladder augmentation.

### Case 2

A 4-year-old boy with omphalocele, exstrophy of the bladder, imperforate anus, and spinal defects (OEIS) complex suffered from short bowel syndrome, high ileostomy output, and urinary leakage via the perineum. As a newborn, the cecum was removed from within the two hemibladders and an end colostomy was fashioned.

Endoscopy revealed severe inflammation of the hindgut, which was felt to be contributing to the secretory diarrhea. The end colostomy originated from the right colon measuring 4 cm in length. Drawing from insights gained in the previously described case, the cecum and residual 4-cm-long hindgut were removed from the fecal stream and utilized as a bladder augment. The cecum was connected to the native bladder with a Mitrofanoff channel fashioned from the native appendix. Due to the limited colonic length, no pull-through procedure was considered, and an end ileostomy was created. Following the surgery, the episodes of bacteremia were resolved as well as the high output from the stoma.

### Case 3

The third patient, described previously, 7 was a 32-week-old female born at 1.7 kg with a giant liver-containing omphalocele that posed a challenge to the standard surgical approach of early closure and conversion to bladder exstrophy. In the neonatal period, the patient drained stool from the cecal plate and urine from the bladder halves, which could be managed well with no fecal diversion.

**Fig. 2:**
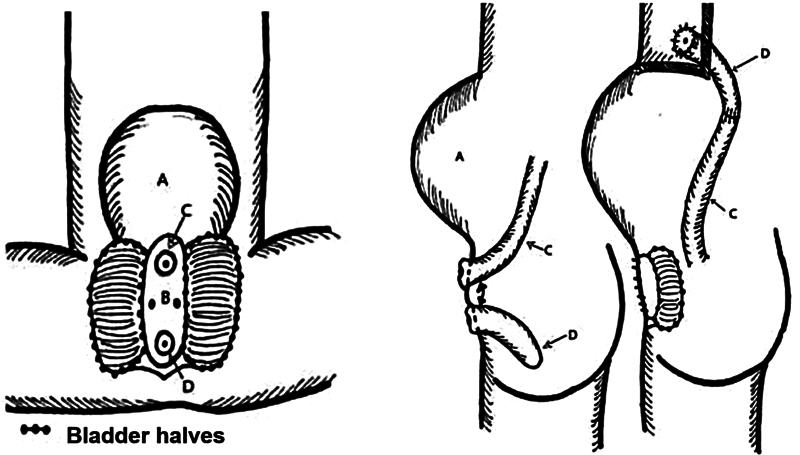
The cecal plate was left untouched between the hemibladders. After cecal plate separation, the ileum was anastomosed with the hindgut. ( **A** ) Omphalocele; ( **B** ) Cecal plate; ( **C** ) Ileum; ( **D** ) Hindgut. (Reproduced from Smith et al. 7 )

**Fig. 3:**
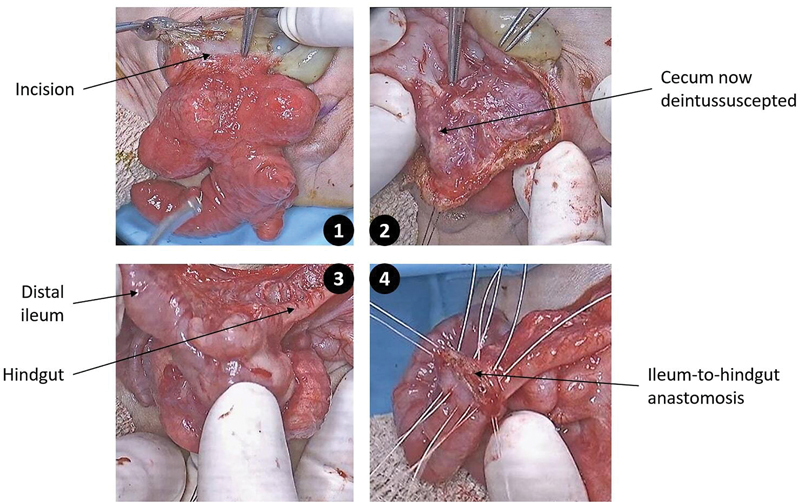
Novel technique of cloacal exstrophy repair. The cecum remains attached to the bladder halves, and the ileum is anastomosed to the hindgut. (This image is provided courtesy of the Children's National Hospital Archives.)

At 4 months of life, the omphalocele sac had fully epithelialized. Given the inability to close the abdominal wall due to the large omphalocele, a unique approach was implemented to bridge the gap between the bladder halves. This involved using a terminal ileum bowel flap, retaining the cecal plate in situ, and effectively autoaugmenting the bladder ( Fig. 2 ). Intraoperative images of the described technique are demonstrated in Fig. 3 . Following the surgery, the patient had a favorable outcome. Enteral feeds were accomplished without symptoms or signs of dumping.

### Case 4

A premature patient born at 33 weeks of gestation, weighing 1.9 kg, presented with omphalocele and cloacal exstrophy. At the time of the initial surgery on day 3 of life, only 2 cm of hindgut was detected. The omphalocele membrane was excised and closed, while the cecum was left in situ between the hemibladders. The 2-cm hindgut was excised, and an end ileostomy was created. At 10 months of age, weighing 7.2 kg, the patient underwent urologic reconstruction with bladder neck closure. The cecal tissue was utilized to form a colovesicostomy, with a stoma for urinary collection. The native vaginas were pulled through and fashioned into a single vagina, while the ileostomy was left in situ. There were no episodes of acidosis recorded.

## Discussion

Addressing cloacal exstrophy presents notable surgical complexities, requiring collaboration from a multidisciplinary surgical team. The traditionally described newborn operation involves separating the cecal plate from the center of the two bladder halves and closing the omphalocele, effectively transitioning the patient to a pure bladder exstrophy, with its subsequent closure occurring at an older age. This approach, seen in the first two presented cases, can lead to significant postoperative complications (bacterial overgrowth and increased ostomy output) related to the cecum's presence in the fecal stream, necessitating later disconnection. Based on these two cases, we propose to leave the cecal plate untouched, within the two hemibladders. This maneuver is technically easier and allows for an ileal to hindgut anastomosis. The cecal plate remains isolated from the fecal stream. The concern noted in the literature of the development of acidosis from urinary absorption by the cecum did not occur, and we know this to not be the case in the vast majority of patients with bladder augmentation either using small bowel or colon. 8 9

Preserving colonic length is vital for achieving appropriate stool consistency for future pull-through and ensuring social continence. 5 6 10 11 However, it is clear from a recent review 12 that very few patients qualify for such a pull-through as they lack adequate colon to produce solid stool. This report differs greatly from previous reports promoting a pull-through for the majority of these patients. 6 Although separating the cecum from the fecal stream gains some digestive tract length, the increase is small. Conversely, employing the cecum for bladder augmentation eliminates the necessity for bowel resection in traditional bladder reconstruction 13 14 and allows for a low-pressure bladder by expanding the volume of the continence reservoir and potentially enhancing urinary function. Disconnection of the cecum from the fecal stream also reduces complications related to bacterial translocation and cecal inflammation, such as increased ostomy output, feeding intolerance, and failure to thrive. Moreover, the newborn operation is technically easier and shorter, if the distal ileum is connected to the hindgut, leaving the cecal plate untouched.

The proposed technique has certain disadvantages. There are concerns regarding the development of acidosis from colonic absorption of urine; however, this did not occur in our patient. This risk is highlighted as a potential drawback of using the bowel in urinary reconstruction regardless of the bowel part used as an augment. 8 While the cecum and right colon have been reported to lead to worse acidosis, this has not been shown to result in worse outcomes. 7 In addition, the technique involves ileocecal valve disruption, which can potentially have implications for digestive processes, and lead to complications such as fecal incontinence. 15

We suggest an alternative approach to managing cloacal exstrophy in newborns by retaining the cecal plate in its original position, connected to the urinary tract and isolated from the fecal stream. This strategy offers potential long-term advantages, including (1) a technically simpler newborn operation, (2) prevention of stasis-related inflammation in the tubularized bowel, and (3) autoaugmentation of the bladder. While acknowledging the risks of acidosis and loss of the ileocecal valve, our findings suggest these concerns may be manageable and do not outweigh the benefits observed.

## Notes

Importance for a Pediatric Surgeon This manuscript introduces a novel approach to the newborn management of cloacal exstrophy by leaving the cecal plate in situ between the hemibladders. This departure from the traditional treatment protocol aims to improve postoperative outcomes by avoiding issues like excessive bacterial overgrowth and increased ostomy output associated with removing the cecum from its original location and including it in the fecal stream.
